# Dynamic frontoparietal flexibility and cognitive dysfunction in schizophrenia: disentangling the roles of symptom burden and childhood trauma

**DOI:** 10.1017/S0033291726103869

**Published:** 2026-04-07

**Authors:** Danqing Huang, Zhening Liu, Wenjian Tan, Michel Sopodenkiewicz, Xiawei Liu, Jun Yang, Feiwen Wang, Weiqing Huang, Jie Yang, Yicheng Long, Lena Palaniyappan

**Affiliations:** 1 Department of Psychiatry, and National Clinical Research Center for Mental Disorders, The Second Xiangya Hospital, Central South University, China; 2 Douglas Mental Health University Institute, Department of Psychiatry, McGill University, Montreal, Canada; 3 Department of Psychology, McGill University, Montreal, Canada

**Keywords:** childhood trauma, fMRI, schizophrenia, temporal variability, triple networks, working memory, resilience, salience network, default mode, resting, task-negative, psychosis, cognition

## Abstract

**Background:**

Working memory (WM) impairment is a core cognitive deficit in schizophrenia, associated with dysfunction of large-scale brain networks, particularly the triple-network system comprising the default mode, frontoparietal, and salience networks. Given the role of environmental risks like childhood trauma (CT) in cognitive deficits, we investigated whether trauma relates to altered triple-network flexibility and WM in schizophrenia.

**Methods:**

We enrolled 190 patients with schizophrenia (SZ) and 117 healthy controls (HCs). Among them, 162 SZ and 99 HCs underwent *n*-back task-based functional magnetic resonance imaging. We computed temporal variability (TV) in the triple-network connectivity, defining ΔTV as the change between 0-back and 2-back conditions. Subgroup comparisons of ΔTV were conducted within each group based on trauma status. Associations of ΔTV with WM performance and clinical symptoms were examined in SZ, followed by mediation analyses testing whether ΔTV mediates the relationship between trauma and WM.

**Results:**

Among HCs, individuals with childhood trauma showed reduced ΔTV across triple-network connections, whereas no such differences appeared in SZ. In SZ, greater ΔTV within the frontoparietal network (FPN) was correlated with lower positive symptom severity (*r* = −0.211, *p*-fdr = 0.046) and better *n*-back target accuracy (*r* = 0.303, *p*-fdr = 0.002). Furthermore, ΔTV within the FPN partially mediated the association between trauma and *n*-back accuracy.

**Conclusions:**

Our findings highlight the central role of FPN flexibility in mediating childhood trauma’s effect on working memory in schizophrenia. This outlines a key pathway through which an early environmental risk (trauma) translates into cognitive and clinical manifestations in schizophrenia.

## Introduction

Schizophrenia is a severe and chronic psychiatric disorder that affects ~1% of the global population and remains a leading cause of long-term disability (Marder & Cannon, 2019). It manifests through a heterogeneous constellation of symptoms, encompassing positive symptoms, negative symptoms, and pervasive cognitive impairments involving attention, working memory (WM), and executive functioning (Lett et al., 2014). While antipsychotic drugs have proven effective in reducing positive symptoms, current antipsychotics have limited effects on cognition (Feber et al., 2025; Keefe et al., 2007). Among these, WM impairment stands out as a central and persistent deficit (Lett et al., 2014): it is evident early in the illness (Nuechterlein, Ventura, Subotnik, & Bartzokis, 2014), endures even when other clinical symptoms have remitted, and strongly predicts clinical (Jenkins, Bodapati, Sharma, & Rosen, 2018) and functional outcomes (Borgan et al., 2021; Fu, Czajkowski, Rund, & Torgalsbøen, 2017) such as occupational attainment and social recovery. Notably, WM impairments remain largely refractory to available treatments, resulting in a persistent illness burden (Park et al., 1999) that may lead to recurrent relapses (Hui et al., 2016) and worsen negative symptoms (Carter et al., 1996; González-Ortega et al., 2013; Nejad et al., 2013; Pantelis et al., 2001). Despite their clinical significance, WM deficits in schizophrenia show only modest and variable improvement following current cognitive and behavioral interventions, highlighting the urgent need to better delineate their underlying neural mechanisms (Lejeune, Northrop, & Kurtz, 2021).

WM refers to the ability to temporarily store, manipulate, and update information in support of goal-directed behavior and serves as a foundation for higher-order cognition (Baddeley, 1992; Hartley & Hitch, 2022). In schizophrenia, WM deficits manifest as difficulties in task switching and coping with complex cognitive demands (Forbes, Carrick, McIntosh, & Lawrie, 2009), and are commonly assessed using paradigms such as the *n*-back task. During WM tasks, extant neuroimaging evidence indicates that patients with schizophrenia show abnormal network-level coordination (Nielsen et al., 2017; Repovš & Barch, 2012; Wang et al., 2024, 2025; Yang et al., 2025). The triple network, which includes the default mode network (DMN), the frontoparietal control network (FPN), and the salience network (SAL), has been proposed as a key systems-level model for understanding WM, as effective performance requires dynamic coordination among these networks (Menon, 2011; Wu & Jiang, 2020). In healthy individuals, WM task performance typically involves the suppression of the DMN and activation of task-positive networks, with the opposing interaction between these systems playing a key role in balancing internal and external attention (Singh & Fawcett, 2008). In contrast, schizophrenia is characterized by disrupted coordination among these large-scale networks (Menon, Palaniyappan, & Supekar, 2023). During the WM task, schizophrenia shows impaired suppression (hyperactivity) of the DMN (Pu et al., 2016; Wang et al., 2022; Wu & Jiang, 2020; Yang et al., 2020), reduced FPN (Greenman et al., 2020; Nielsen et al., 2017; Pu et al., 2016) coupling, and disrupted interactions among DMN, FPN, and SAL (Braun et al., 2021; Luo et al., 2020; Pu et al., 2016; Zhou et al., 2018). Moreover, while healthy individuals efficiently deactivate DMN regions and recruit task-relevant hubs, patients often fail to suppress the DMN and instead engage task-irrelevant regions during *n*-back tasks (Huang et al., 2019; Pu et al., 2016; Williams et al., 2023; Wu & Jiang, 2020). Taken together, these alterations indicate inefficient allocation of neural resources, likely due to reconfiguration of connectivity patterns during WM, leading to poorer performance. Against this backdrop, we turn to the FPN, the principal executor of control signals during WM within the triple-network architecture.

Most prior WM studies in schizophrenia have relied on static functional connectivity (sFC), which assumes stationarity of signals and fails to capture the time-varying reconfiguration of large-scale networks. Functional connectivity, however, is not stationary but rather fluctuates over time (Chang & Glover, 2010; Hutchison et al., 2013). These fluctuations (Long, Liu, & Liu, 2023; Preti, Bolton, & Van De Ville, 2017) are often referred to as dynamic functional connectivity (dFC) and offer valuable insights into brain organization by revealing subtle temporal dynamics (Allen et al., 2014; Fu et al., 2021; Hutchison et al., 2013; Sakoğlu et al., 2010) that static FC analyses are unable to capture. Recent work has therefore shifted toward dynamic measures, including state occupancy/transition rates (Braun et al., 2021; Wang et al., 2025), dFC (Cassidy et al., 2016), and graph-based variability metrics (Cheng et al., 2025; Dimitriadis et al., 2021). These studies consistently demonstrate abnormal frontoparietal dynamics in schizophrenia during WM tasks, such as reduced stability of FPN-DMN antagonistic states (Wang et al., 2025), blunted load-dependent modulation within FPN (Nielsen et al., 2017), and inefficient reallocation of resources via salience network driven switching (Luo et al., 2020).

Temporal variability (TV) (Long et al., 2020; Long et al., 2020; Sun et al., 2019; Zhang et al., 2016) is a dynamic connectivity metric that focuses on the changing configurations in functional connectivity strength across time windows. In schizophrenia, TV has been primarily examined in the resting state. Patients consistently show increased variability within large-scale networks such as the DMN and SAL (Long, Liu, et al., 2020; Rolls, Cheng, & Feng, 2021), and these alterations have been associated with greater symptom severity (Long, Liu, et al., 2020) and cognitive impairments (Yue et al., 2018; Zhang et al., 2016. In this study, we estimate TV for the first time during a WM paradigm in schizophrenia, as a metric to assess load-dependent network flexibility (ΔTV, defined as the difference in variability between high- and low-load WM conditions) during task performance.

Aberrant brain network properties are increasingly recognized to be shaped by environment factors, with childhood trauma (CT) emerging as a major environmental risk factor for psychosis (Kraan et al., 2015). Evidence suggests that exposure to CT substantially elevates the risk of schizophrenia, with some studies estimating up to a threefold increase compared with nonexposed individuals (Aas et al., 2016;Croft et al., 2019; De-Nardin et al., 2022). This risk follows a dose–response pattern, whereby earlier, more severe, and more frequent trauma confers proportionally greater vulnerability (Croft et al., 2019; De-Nardin et al., 2022). Beyond illness onset, CT has been linked to worse clinical outcomes, including greater symptom severity (Ruby et al., 2017), poorer treatment response (Kilian et al., 2020), and persistent cognitive impairments (S. Kilian et al., 2018; Wells et al., 2020) such as deficits in WM (Popovic et al., 2019). Recent evidence also indicates that CT may exert long-lasting neurodevelopmental effects, altering large-scale brain networks that support cognition and thereby shaping trajectories of vulnerability to psychosis (Lipner et al., 2023; Liu et al., 2021). Notably, converging evidence suggests that childhood trauma affects prefrontal and frontoparietal control systems, which are highly sensitive to developmental adversity and are central to executive control and WM (Gerin et al., 2023; Philip et al., 2016, 2013).

**Figure 1.**
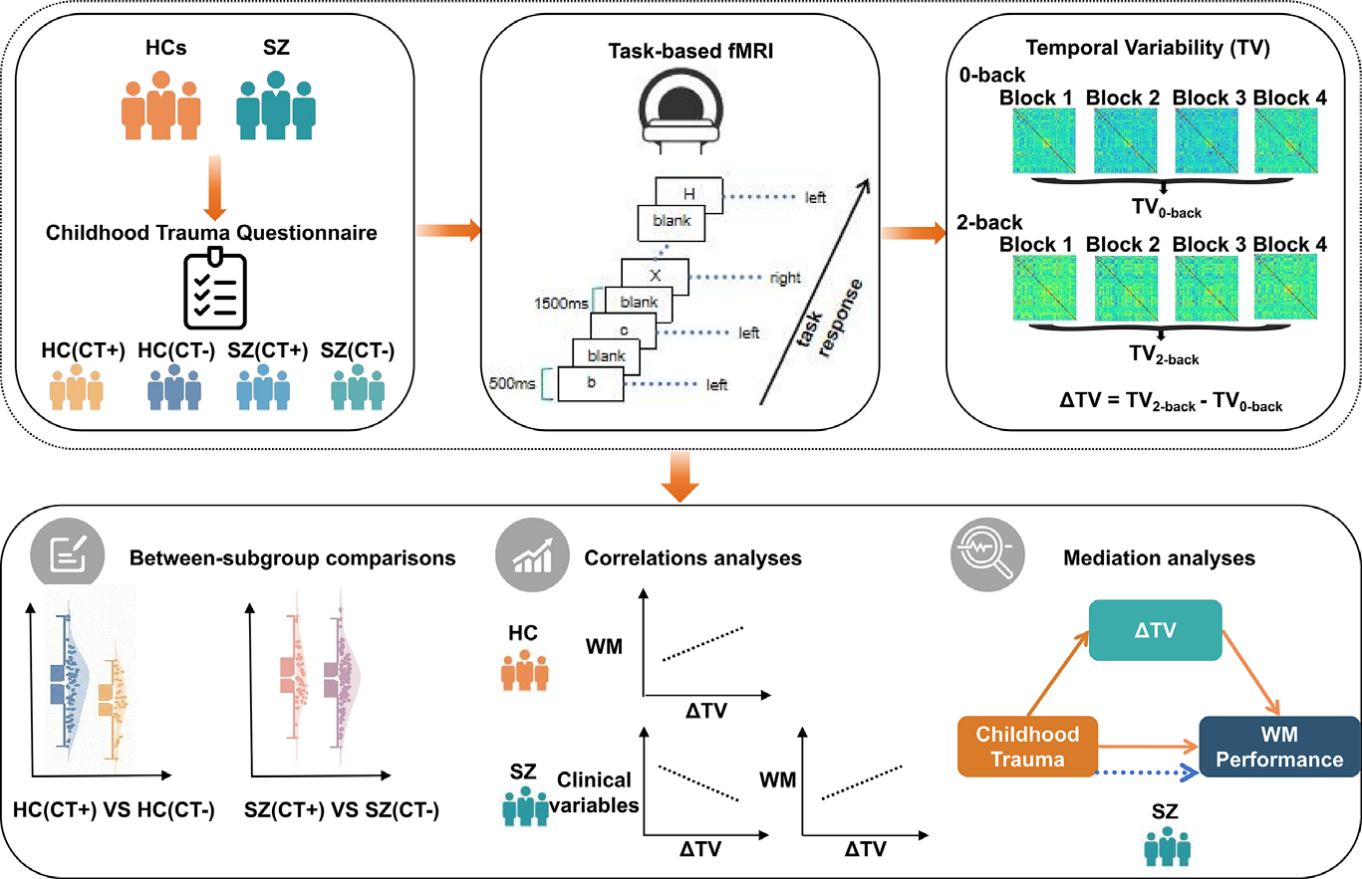
Schematic illustration of the study design and analytic pipeline. *Note*: *n*-back task-based fMRI data were collected from patients with SZ and HCs. Temporal variability (TV) of dynamic functional connectivity (dFC) was assessed under different task conditions, and ΔTV was calculated as the difference in TV between the 2-back and 0-back conditions. Participants were further stratified by the presence or absence of childhood trauma, and subgroup differences in ΔTV, as well as associations with clinical symptoms and cognitive performance, were examined.

Against this background, this study integrates CT, TV, and WM performance into a unified framework. We hypothesize that CT will affect WM via altered TV. Specifically, we focus on the triple-network systems (DMN, FPN, and SAL), with particular emphasis on the FPN as the core control hub that is likely to be disrupted in the presence of childhood trauma. Based on prior evidence and theoretical considerations, we aimed to: (1) determine whether childhood trauma affects triple-network TV in patients and control subjects; (2) examine whether ΔTV within the FPN affects WM performance and symptom burden in patients with schizophrenia; and (3) test whether altered TV of the FPN mediates the relationship between trauma exposure and WM deficits in schizophrenia. Establishing these relationships will clarify the neurophysiological mechanism by which exposure to CT may affect core cognitive functions and symptoms in schizophrenia. A schematic overview of the research design is shown in Figure 1.

## Materials and methods

### Participants

A total of 190 patients with schizophrenia were recruited from the Second Xiangya Hospital of Central South University. A total of 117 healthy controls (HCs) were recruited from Changsha and its surrounding areas. All participants underwent brief, unstructured interviews conducted by psychiatrists and provided written informed consent before taking part in the study. The study was approved by the Ethics Committee of the Second Xiangya Hospital of Central South University. All procedures were carried out in strict accordance with the Declaration of Helsinki. Inclusion criteria for the patient group were right-handedness and a confirmed diagnosis of schizophrenia according to DSM-5. Exclusion criteria for patients were: (1) a primary psychiatric diagnosis other than schizophrenia; (2) a history of serious physical illness or neurological conditions; (3) current or past substance abuse or dependence (except for nicotine use); and (4) metal implants (e.g. electronic devices) or other contraindications to MRI. HCs were screened using the same general exclusion criteria, with the additional requirement that none met DSM-5 criteria for any psychiatric illness.

### Clinical assessment

The severity of clinical syndromes in schizophrenia was evaluated by trained psychiatrists using the Positive and Negative Syndrome Scale (PANSS) (Kay, Fiszbein, & Opler, 1987). The PANSS comprises 30 items divided into three subscales: positive symptoms, negative symptoms, and general psychopathology. It assesses symptom severity over the past month, with each item rated on a 7-point scale and offers a thorough evaluation of the patient’s mental state.

Following previous research (Shafer & Dazzi, 2019), we derived PANSS five-factor scores, which provide a more nuanced dimensional structure. These include negative symptoms, positive symptoms, disorganization (often termed cognitive), excitement/activity (resistance), and affect (often termed depression-anxiety).

### Assessment of childhood trauma

Childhood trauma exposure was assessed using the Chinese version of the Childhood Trauma Questionnaire (CTQ) (Bernstein et al., 2003), a validated (Zhao et al., 2005) self-reported instrument that has been widely used in previous research (Huang et al., 2021; Li et al., 2014; Lu et al., 2020; Yang et al., 2022). The CTQ consists of 25 clinical items organized into five subscales: emotional abuse, physical abuse, sexual abuse, emotional neglect, and physical neglect, each consisting of five items. Responses are scored on a 5-point Likert scale ranging from 1 (*never*) to 5 (*very often*), reflecting the frequency of the reported experiences. Based on the cutoff criteria used in earlier studies (Huang et al., 2021; Lu et al., 2016; Lu et al., 2017; Yang et al., 2017), participants were considered to have experienced childhood trauma if their scores exceeded the predefined thresholds for any subscale. The cutoffs were as follows: emotional abuse (≥13), physical abuse (≥10), sexual abuse (≥8), emotional neglect (≥15), or physical neglect (≥10).

### Imaging data acquisition and preprocessing

The MRI imaging data were acquired from a Siemens 3-T scanner with the following parameters: 32 slices, 64 × 64 matrix, 5 mm slice thickness with no inter-slice gap, 90° flip angle, field of view of 240 × 240 mm^2^, repetition time (TR) of 2000 ms, echo time (TE) of 30 ms, and a total of 253 time points collected.

Preprocessing of the data was performed using the DPARSF software (Chao-Gan & Yu-Feng, 2010; Yan, Wang, Zuo, & Zang, 2016) (http://rfmri.org/DPARSF), adhering to a standardized processing pipeline. The initial five volumes were discarded, after which slice-timing correction, motion realignment, spatial normalization, and temporal band-pass filtering (0.01–0.10 Hz) were applied. To control for nuisance signals, covariates including white matter and cerebrospinal fluid signals were regressed out. Subsequently, experienced researchers manually evaluated image quality. To ensure the reliability of the data, mean framewise displacement (FD) was computed for each subject. Datasets exhibiting excessive head motion (mean FD > 0.2 mm) or poor image quality, as determined by visual inspection, were excluded from further analyses. After image quality control, a total of 162 individuals with schizophrenia and 99 HCs were included in the study. For each participant, time series were extracted from 264 regions of interest (ROIs) based on Power et al.’s (2011) atlas. These ROIs were assigned to nine functional networks according to previous publications: default mode network (DMN), salience network (SAL), visual network (VIS), subcortical network (SUB), auditory network (AUD), frontoparietal network (FPN), cingulo-opercular network (CON), sensorimotor network (SM), and attention network (ATT).

### n-back task and performance

We employed the ‘*n*-back’ paradigm to assess WM function, utilizing both 0-back and 2-back conditions in this study. In the 0-back task, participants were instructed to press the right button whenever the letter ‘X’ or ‘x’ appeared, and the left button for all other letters. In the 2-back task, they were required to press the right button when the current letter matched the one presented two trials earlier, regardless of case (e.g. ‘D’ and ‘d’ were considered the same); otherwise, they pressed the left button.

The entire task-based fMRI session consisted of four 40-second blocks of the 0-back task and four 40-second blocks of the 2-back task. A 2-second instruction cue was presented before each task block, and a 20-second fixation period followed it. Participants were asked to fixate on a central crosshair during the fixation. The cue and fixation were not part of the task blocks. All stimuli were white letters presented at the center of the screen, each displayed for 500 ms with an interstimulus interval of 1500 ms. The stimulus sequence was pseudorandomized throughout the session.

To assess the performance on the *n*-back task, we calculated both accuracy (ACC) and response time (RT) measures. Specifically, six indices were derived: target accuracy (target ACC), overall accuracy in the 0-back condition (0-back ACC), overall accuracy in the 2-back condition (2-back ACC), as well as response times for target trials (target RT), 0-back trials (0-back RT), and 2-back trials (2-back RT). Target ACC was defined as the proportion of correct responses to target stimuli. Overall ACC in the 0-back and 2-back conditions reflected the proportion of correct responses across all trials within each condition, including both target and nontarget trials. Response times were calculated only for correct trials and averaged within each condition.

### Temporal variability

The method for calculating TV followed established procedures in previous literature (Long, Liu, et al., 2020; Sun et al., 2022; Zhang et al., 2016. To evaluate the TV of dFC under different task conditions, we segmented the preprocessed ROI time series to retain only the task execution periods (i.e. blocks). Each block lasted 40 seconds (20 TRs); task instruction and fixation periods were excluded. This segmentation produced four blocks for the 0-back condition and four for the 2-back condition.

For each block, pairwise Pearson correlation coefficients between all ROIs were computed to construct a dFC matrix. The TV of dFC was then calculated across blocks for each condition at three spatial scales: intra-network, inter-network, and whole-brain. Specifically, the intra-network TV for a given network (e.g. the DMN) was defined as:

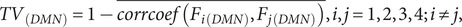

where *F_i_* (DMN) is a vector representing the dFC between all ROI pairs within the DMN in the *i*th block. Similarly, the inter-network TV of dFC between two networks (e.g. the DMN and SAL) was computed as:

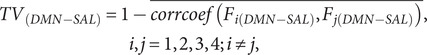

where *F_i_* (DMN-SAL) is a vector representing the dFC between all ROIs in the DMN and all ROIs in the SAL in the *i*th block, where the overline denotes the average of the Pearson correlation coefficients computed across all unique block pairs. The TVs for all other individual networks and network pairs were calculated using the same method. Consequently, for each condition (0-back and 2-back), the above analyses produced a total of 9 intra-network TV values (1 per network) and 36 inter-network TV values (1 per unique network pair).

To further characterize task-related changes in TV of dFC, we computed the difference in TV between the 2-back and 0-back conditions for each participant. This difference metric, denoted as ΔTV, was calculated as:

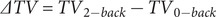

where TV_2-back_ and TV_0-back_ represent the TV values derived from the 2-back and 0-back tasks, respectively. The ΔTV thus reflects the individual-level modulation of dynamic brain connectivity variability induced by increased WM load. The ΔTV was calculated for all intra-network and inter-network TVs.

### Statistics

In this study, based on the classification criteria described above, participants with schizophrenia and HCs were further stratified into subgroups with and without a history of childhood trauma. This resulted in 110 schizophrenia patients with childhood trauma and 52 without, as well as 34 HCs with childhood trauma and 65 without.

To compare demographic characteristics between groups, independent samples *t*-tests were used for age, years of education, head motion (measured by mean framewise displacement, FD), and CTQ score, while chi-square tests were conducted for sex distribution.

In the HC group, we compared ΔTV between individuals with and without childhood trauma. The analysis focused on the triple network, covering both within-network (DMN, SAL, and FPN) and between-network (DMN-SAL, DMN-FPN, and SAL-FPN) connections. An analysis of covariance (ANCOVA) was performed with sex, age, and head motion as covariates. The same ANCOVA was performed in the schizophrenia group to compare those with and without trauma. Results were considered significant at *p* < 0.05 after FDR correction, which was applied across the six ΔTV measures within each group separately.

In patients with schizophrenia, partial correlation analyses were performed to examine the associations between the six previously mentioned ΔTV and clinical symptoms, including PANSS total score, PANSS subscale scores, and PANSS five-factor scores, while controlling for age, sex, and head motion. Additionally, partial correlation analyses were conducted in both the schizophrenia and HC groups to assess the associations between these ΔTV and *n*-back task performance, controlling for the same covariates. All correlation results were corrected for multiple comparisons using FDR correction, applied across the six ΔTV measures for each outcome variable within each group, with *p* < 0.05 considered statistically significant.

To examine whether ΔTV in the triple network mediates the association between childhood trauma and *n*-back performance in patients with schizophrenia, we conducted six separate mediation analyses using the PROCESS macro for SPSS, specifying Model 4. In each model, the total score of the CTQ was used as the independent variable (X), and *n*-back target accuracy was entered as the dependent variable (Y). Each of the six ΔTV related to the triple network was entered separately as a mediator (M). Sex, age, and mean FD were included as covariates. The significance of indirect effects was assessed using nonparametric bootstrapping (5,000 resamples), with statistical significance determined by 95% bootstrap confidence intervals.

### Supplementary analyses

To further explore group-level differences in temporal variability between patients with schizophrenia and HCs, we conducted additional analyses using a subsample matched for age and sex (115 SZ and 85 HCs). Specifically, ΔTV, TV_0-back_, and TV_2-back_ were compared across all functional networks using ANCOVA, with sex, age, and mean FD included as covariates.

## Results

### Demographic, clinical, head motion, and CTQ characteristics

As shown in Table 1, within both the SZ and HC groups, there were no significant differences in age, sex, years of education, or head motion between individuals with and without childhood trauma.

**Table 1.** Comparison of demographic, clinical, head motion, and CTQ characteristics between CT+ and CT− individuals within the SZ and HC groups

SZ group	CT+ (*n* = 110), mean ± SD	CT− (*n* = 52), mean ± SD	Group comparisons
Age (years)	17.81 ± 2.84	18.08 ± 3.28	*t* = −0.533, *p* = 0.595
Education (years)	10.77 ± 2.33	11.03 ± 2.22	*t* = −0.674, *p* = 0.501
Sex (male/female)	63/47	27/25	*χ*^2^ = 0.409, *p* = 0.522
Mean FD (mm)	0.077 ± 0.045	0.067 ± 0.041	*t* = 1.306, *p* = 0.193
PANSS score	71.11 ± 22.32	63.29 ± 21.24	*t* = 2.111, *p* = 0.036
CTQ scores	59.40 ± 10.30	44.02 ± 4.35	*t* = 13.347, *p* < 0.001

HC group	CT+ (*n* = 34), mean ± SD	CT− (*n* = 65), mean ± SD	Group comparisons
Age (years)	20.53 ± 3.69	20.98 ± 3.73	*t* = −0.579, *p* = 0.564
Education (years)	13.52 ± 3.14	13.29 ± 3.30	*t* = 0.335, *p* = 0.738
Sex (male/female)	17/17	29/36	*χ*^2^ = 0.260, *p* = 0.610
Mean FD (mm)	0.058 ± 0.025	0.063 ± 0.034	*t* = −0.739, *p* = 0.420
CTQ scores	52.47 ± 6.53	43.22 ± 3.81	*t* = 7.617, *p* < 0.001

*Note:* SZ, schizophrenia; HC, healthy control; CTQ, Childhood Trauma Questionnaire; CT+, individuals with childhood trauma; CT−, individuals without childhood trauma; SD, standard deviation; Mean FD, mean framewise displacement. One patient in the CTQ+ group did not complete the PANSS, resulting in a slightly reduced sample size (*n* = 109) for that variable.

A significant difference in total PANSS scores was observed between schizophrenia patients with and without childhood trauma, with the CT+ group showing higher scores than the CT− group (*F* = 0.279, *p* = 0.036).

### ANCOVA results for ΔTV differences within groups

**Figure 2.**
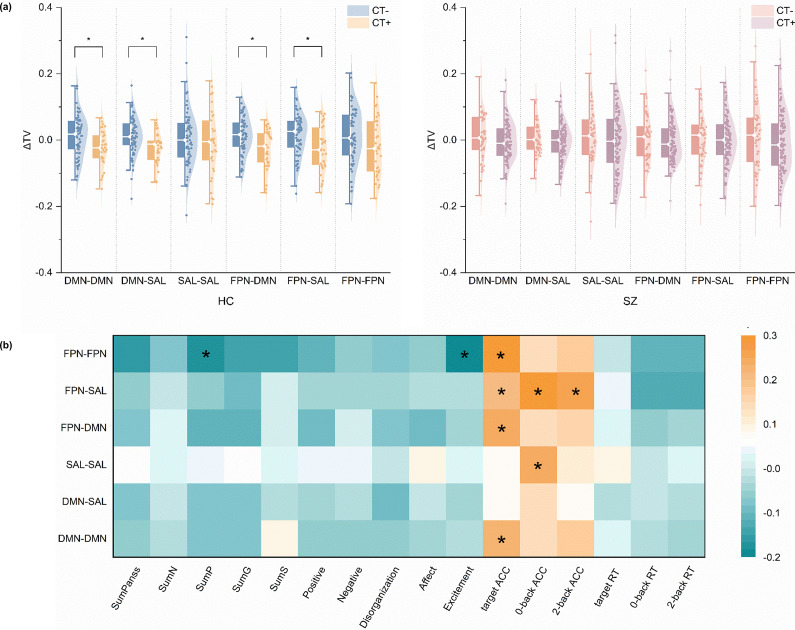
(a) Within-group effects of childhood trauma on ΔTV across the triple network: left, HCs (CT+ vs. CT−); right, SZ (CT+ vs. CT−). (b) Partial correlation results between ΔTV and clinical and task performance in schizophrenia. *Note*: ΔTV, change in temporal variability of dynamic functional connectivity between the 2-back and 0-back conditions; HC, healthy control; SZ, schizophrenia; CT+, individuals with childhood trauma; CT−, individuals without childhood trauma; DMN, default mode network; SAL, salience network; FPN, frontoparietal network; SumN, negative symptoms; SumP, positive symptoms; SumG, general psychopathology; SumS, specific symptoms; ACC, accuracy; RT, response time; ‘*’ indicates corrected *p* < 0.05.

All *p*-values reported are corrected for multiple comparisons using the false discovery rate (fdr) method. As shown in Table 2 and Figure 2, within the HC group, ANCOVA revealed significant differences in ΔTV between participants with and without childhood trauma for the network with DMN (*F* = 7.641, *p*-fdr = 0.011), between the DMN and SAL (*F* = 8.706, *p*-fdr = 0.011), between the FPN and DMN (*F* = 10.246, *p*-fdr = 0.011), and between FPN and SAL (*F* = 7.981, *p*-fdr = 0.011) networks, with CT– individuals showing positive mean ΔTV values (higher flexibility with 2-back load vs. 0-back) and CT+ individuals showing negative mean values (lower flexibility with 2-back load vs. 0-back). No significant within-network changes were observed for SAL or FPN. All analyses controlled for age, sex, and head motion. No significant ΔTV differences were observed between CT+ and CT− individuals with schizophrenia across all triple-network pairs, indicating that the connectivity-related effects of trauma were not prominent in the presence of schizophrenia.

**Table 2.** ANCOVA results for ΔTV differences within HC

ΔTV	CT+ (mean ± SD)	CT− (Mean ± SD)	*F*	*p*	*p*-fdr
DMN-DMN	−0.004 ± 0.058	0.030 ± 0.067	7.641	0.007	0.011
DMN-SAL	−0.018 ± 0.047	0.016 ± 0.060	8.706	0.004	0.011
SAL-SAL	−0.002 ± 0.097	0.013 ± 0.088	0.571	0.452	0.452
FPN-DMN	−0.012 ± 0.062	0.026 ± 0.058	10.246	0.002	0.011
FPN-SAL	−0.014 ± 0.069	0.027 ± 0.064	7.981	0.006	0.011
FPN-FPN	0.005 ± 0.087	0.034 ± 0.086	2.858	0.094	0.113

*Note:* ANCOVA, analysis of covariance; ΔTV, change in temporal variability of dynamic functional connectivity between the 2-back and 0-back conditions; HC, healthy control; CT+, individuals with childhood trauma; CT−, individuals without childhood trauma; SD, standard deviation; fdr, false discovery rate; DMN, default mode network; SAL, salience network; FPN, frontoparietal network.

### Partial correlation analysis

As shown in Figure 2, partial correlation analyses controlling for age, sex, and head movement revealed several significant associations between ΔTV and both clinical and task performance variables within the schizophrenia group. Specifically, increased ΔTV within the FPN (i.e. higher flexibility at 2-back state) was significantly associated with lower PANSS positive symptoms (*r* = −0.211, *p*-fdr = 0.046) and lower excitement symptoms (*r* = −0.246, *p*-fdr = 0.012) based on the PANSS five-factor model, while showing a positive association with target accuracy (*r* = 0.303, *p*-fdr = 0.002) on the *n*-back task. ΔTV between the FPN and SAL network (i.e. higher 2-back related flexibility) was positively correlated with target accuracy (*r* = 0.211, *p*-fdr = 0.024), 0-back accuracy (*r* = 0.309, *p*-fdr = 0.002), and 2-back accuracy (*r* = 0.26, *p*-fdr = 0.018). Furthermore, both ΔTV between the FPN and DMN (*r* = 0.242, *p*-fdr = 0.015) and within DMN (*r* = 0.228, *p*-fdr = 0.018) were positively associated with target accuracy. ΔTV within the SAL network was positively correlated with 0-back accuracy (*r* = 0.236, *p*-fdr = 0.021).

### Mediation analyses

**Figure 3.**
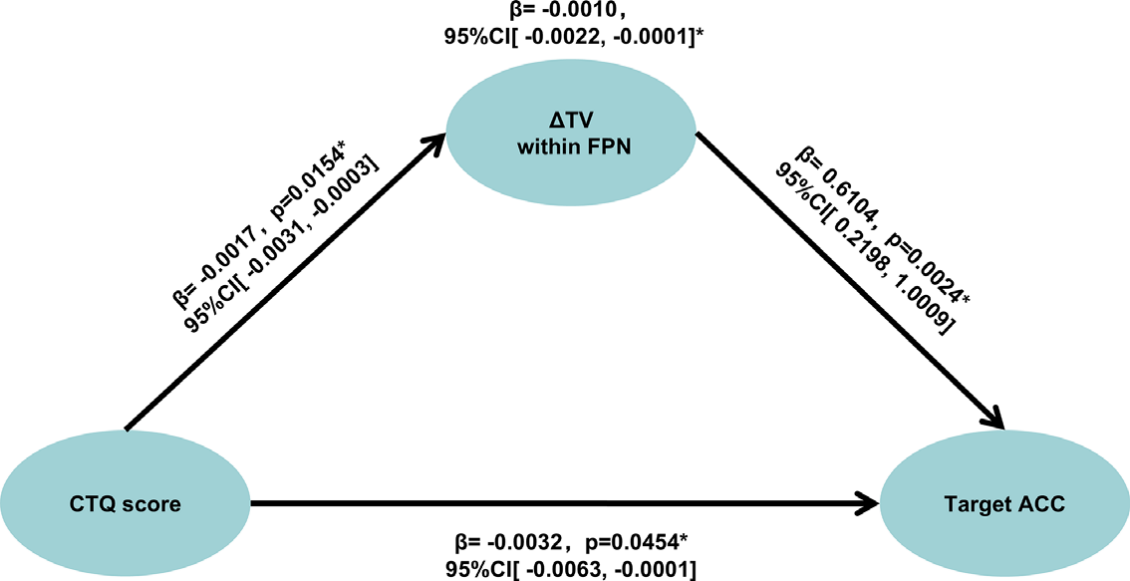
Mediation effect among CTQ score, ΔTV within the FPN, and target accuracy. *Note*: ΔTV, change in temporal variability of dynamic functional connectivity between the 2-back and 0-back conditions; FPN, frontoparietal network; CTQ, Childhood Trauma Questionnaire; ACC, accuracy.

Given the observed associations between ΔTV, childhood trauma in the absence of schizophrenia, and *n*-back performance in schizophrenia, mediation analyses were performed to test potential indirect effects in schizophrenia. As shown in Figure 3, higher CTQ scores were associated with lower target accuracy (*β* = −0.0032, *p* = 0.045) and reduced ΔTV within the FPN (*β* = −0.0017, *p* = 0.015). In turn, greater ΔTV within the FPN predicted better target accuracy (*β* = 0.6104, *p* = 0.002). The indirect effect of CTQ on target accuracy through ΔTV within the FPN was significant (*β* = −0.0010, 95% CI: [−0.0022, −0.0001]), indicating that childhood trauma influenced WM performance in schizophrenia, in part, via altered ΔTV within the FPN.

### Supplementary analyses

To further examine the spatial distribution of load-specific TV changes, we conducted ANCOVA comparing schizophrenia and HC groups on ΔTV, TV_0-back_, and TV_2-back_, controlling for age, sex, and head motion. As summarized in Supplementary Table S1, patients with schizophrenia exhibited significantly higher temporal variability than HCs across multiple network pairs, including between the VIS and DMN, between the VIS and AUD, between the visual and CON, and between the VIS and SM. These findings suggest that abnormal temporal variability during the 2-back task in schizophrenia extends beyond triple-network regions to widespread circuits involving the visual network and its interactions with higher-order control and sensory systems. In addition, a single between-network connection between VIS and AUD showed a significant group difference in ΔTV. Except for the connections reported in Supplementary Tables S1 and S2, all other tested ΔTV, TV_0-back_, and TV_2-back_ connections did not show significant differences between the schizophrenia and HC groups after FDR correction.

## Discussion

In HCs, individuals with childhood trauma (CT+) showed significantly lower ΔTV in triple-network connections than those without trauma exposure (CT−) within DMN, DMN-SAL, FPN-DMN, and FPN-SAL), indicating reduced load-dependent flexibility with CT exposure. On average, CT+ participants exhibited negative ΔTV values, whereas CT− participants showed positive values, indicating that early adversity is linked to diminished network flexibility across cognitive load conditions. This pattern suggests that early adversity reduces the brain’s capacity to flexibly reconfigure large-scale networks. In schizophrenia, while trauma did not have a group-level effect on triple-network TV, a higher load of trauma affected WM performance by disrupting frontoparietal TV, and was associated with a higher burden of positive and excitement symptoms.

Converging evidence supports this interpretation. Previous research has shown that childhood maltreatment alters connectivity within and between large-scale networks relevant for WM (Philip et al., 2016, 2013), compromising adaptive coordination for cognitive control. However, task-based neuroimaging studies directly testing the impact of childhood trauma remain relatively rare. By contrast, resting-state studies consistently report adversity-related alterations in intrinsic functional connectivity, especially between the DMN, SAL, and FPN. For example, a recent systematic review reported that childhood maltreatment is linked to reduced resting-state connectivity between the anterior insula and the dorsal anterior cingulate cortex, alongside heightened amygdala coupling within the triple network (Gerin et al., 2023). Additionally, our previous study (D. Huang et al., 2021) found that higher CTQ scores predicted reduced temporal stability across large-scale networks. These alterations were mostly found in the FPN and DMN, as well as in CON and VIS. These results suggest that early-life adversity reduces the stability and flexibility of key intrinsic networks, even in the absence of task demands. This finding aligns with our observation of reduced ΔTV during WM load in HCs.

Why are these effects detectable in healthy individuals but muted in schizophrenia? Mechanistically, childhood adversity can induce enduring neurodevelopmental alterations that disrupt network maturation (Murphy et al., 2022). This may disproportionately impact late-maturing frontoparietal and salience systems (Tomoda et al., 2024). By contrast, schizophrenia is characterized by baseline disruptions in triple-network dynamics, including inefficient DMN suppression during cognition and weakened FPN coupling. These disease-related dysconnectivity may overshadow incremental trauma effects (a ceiling/occlusion scenario) (Menon et al., 2023). This interpretation aligns with triple-network models and recent work emphasizing dysregulated dynamics during WM in schizophrenia (Supekar et al., 2019; Wang et al., 2025).

In patients with schizophrenia, higher ΔTV within the FPN was associated with lower PANSS positive scores and lower excitement scores, while also relating to better *n*-back target accuracy (age, sex, and head motion controlled). Because ΔTV indexes load-dependent change in temporal variability from 0-back to 2-back, this pattern indicates that a more adaptive increase in FPN variability under higher cognitive load (i.e. larger ΔTV) is linked to reduced positive/excitatory symptom burden and improved performance.

Our mediation analysis demonstrated that ΔTV within the FPN partially mediates the association between CT and WM performance in schizophrenia. Specifically, higher CTQ scores were associated with reduced ΔTV, which in turn predicted poorer target accuracy. This pattern suggests that trauma diminishes the capacity of FPN to flexibly adjust under cognitive load, thereby constraining executive resource allocation, underscoring the vulnerability of this network to early-life stress. Thus, reduced ΔTV constitutes a partially mediating pathway through which trauma relates to WM deficits.

There are several limitations in our study. First, the cross-sectional design limits inferences about temporal precedence and causality. Second, CT was assessed via retrospective self-report (CTQ), which is susceptible to recall bias and current state influences. Third, because the *n*-back task included only two load levels (0- and 2-back), we were unable to examine brain-state characteristics under higher cognitive loads. The present cohort primarily consisted of individuals with early-onset, first-episode schizophrenia, which has been described in our previous work. Although this characteristic may, to some extent, reduce the confounding effects of long-term disease progression, the effect of antipsychotic medication use, and untreated illness duration cannot be ruled out in the current study. Longitudinal studies that include medication-naive individuals or patients at early stages of treatment will be necessary to further disentangle the respective contributions of illness-related factors, treatment effects, and network dynamic alterations. In addition, the prevalence of childhood trauma differed between patients with schizophrenia and HCs. Although this difference may have influenced group-level characteristics, our primary analyses examined trauma effects using within-group stratification (CT+ vs. CT−), which helps to mitigate the impact of unequal trauma prevalence across diagnostic groups.

Quantifying load-dependent temporal variability offers a systems-level perspective on WM dysfunction in schizophrenia. Demand-sensitive FPN flexibility, together with its coordination with the DMN and SAL, appears to link CT to cognitive inefficiency. The mediation result situates reduced FPN ΔTV on the pathway from early adversity to poorer performance of WM, consistent with a mechanism in which insufficient flexibility leads to inefficient resource allocation. These findings delineate a new mechanistic pathway, whereby early adversity impairs WM through disruptions of dynamic brain-network function.

To conclude, we underscore the central role of dynamic frontoparietal flexibility in mediating the effects of childhood trauma on WM performance in schizophrenia. By quantifying load-dependent temporal variability (ΔTV), we reveal a systems-level mechanism linking early adversity to cognitive inefficiency. Importantly, reduced ΔTV within the FPN may reflect a failure to adapt under cognitive load, a deficit that can be targeted with neuromodulation or psychotherapeutic means. These results extend prior connectivity and controllability studies by identifying ΔTV as a sensitive marker of executive dysfunction and trauma-related vulnerability. Taken together, our study delineates a novel mechanistic pathway through which early-life stress disrupts cognitive control via impaired dynamic network modulation, offering new targets for intervention and the development of mechanistic markers of environmental risks in schizophrenia.

## Supporting information

10.1017/S0033291726103869.sm001 Huang et al. supplementary material Huang et al. supplementary material

## Supplementary material

The supplementary material for this article can be found at http://doi.org/10.1017/S0033291726103869.
