# Supplementary material for: Dynamic frontoparietal flexibility and cognitive dysfunction in schizophrenia: disentangling the roles of symptom burden and childhood trauma
Source: Psychol Med. 2026 Apr 7;56:e93. doi: 10.1017/S0033291726103869 (PMC13079207; doi:10.1017/S0033291726103869)
Supplement: Huang et al. supplementary material [file S0033291726103869sup001.docx]

**Table S1.** ANCOVA results for temporal variability during the 2-back task between matched schizophrenia and healthy control groups

| TV_2-back_ | SZ (Mean ± SD) | HC (Mean ± SD) | F | | p | | p-fdr | |
| --- | --- | --- | --- | --- | --- | --- | --- | --- |
| VIS -DMN | 0.911 ± 0.049 | 0.883 ± 0.054 | 14.581 | <.001 | | 0.004 | |  |
| VIS - AUD | 0.948 ± 0.057 | 0.918 ± 0.061 | 15.670 | <.001 | | 0.004 | |  |
| VIS - CON | 0.934 ± 0.053 | 0.907 ± 0.072 | 10.161 | 0.002 | | 0.019 | |  |
| VIS - SM | 0.911 ± 0.058 | 0.881 ± 0.068 | 13.130 | <.001 | | 0.006 | |  |

ANCOVA, analysis of covariance; TV, temporal variability; SZ, schizophrenia; HC, healthy control; SD, Standard Deviation; fdr, false discovery rate; VIS, visual network; DMN, default mode network; AUD, auditory network; CON, cinguloopercular network; SM, sensorimotor network.

**Table S2.** ANCOVA results for ΔTV between matched schizophrenia and healthy control groups

| ΔTV | SZ (Mean ± SD) | HC (Mean ± SD) | F | | p | | p-fdr | |
| --- | --- | --- | --- | --- | --- | --- | --- | --- |
| VIS -AUD | -0.003 ± 0.069 | -0.037 ± 0.066 | 11.664 | <.001 | | 0.035 | |  |

ANCOVA, analysis of covariance; TV, temporal variability; ΔTV, change in temporal variability of dynamic functional connectivity between the 2-back and 0-back conditions; SZ, schizophrenia; HC, healthy control; SD, Standard Deviation; fdr, false discovery rate; VIS, visual network; DMN, default mode network; AUD, auditory network; CON, cinguloopercular network; SM, sensorimotor network.
